# Magnetic resonance detects changes in phosphocholine associated with *Ras* activation and inhibition in NIH 3T3 cells

**DOI:** 10.1054/bjoc.2000.1663

**Published:** 2001-03

**Authors:** S M Ronen, L E Jackson, M Beloueche, M O Leach

**Affiliations:** Cancer Research Campaign (CRC) Clinical Magnetic Resonance Research Group, Institute of Cancer Research, Royal Marsden Hospital, Downs Road,Sutton, Surrey, SM2 5PT, UK

**Keywords:** magnetic resonance, ^31^P spectroscopy, phosphocholine, *Ras*, NIH 3T3

## Abstract

*Ras* is frequently mutated in cancer, and novel therapies are being developed to target Ras signalling. To identify non-invasive surrogate markers of Ras activation and inhibition, we used^31^P magnetic resonance spectroscopy (MRS) and investigated NIH 3T3 cells compared to a mutant *ras* transfected counterpart. The MR spectra indicated that phosphocholine (PC) levels increased significantly from 3 ± 2 fmol cell^−1^in NIH 3T3 cells to 13 ± 4  fmol cell^−1^in the transfected cells. The PC/NTP ratio increased significantly from 0.3 ± 0.1 to 0.7 ± 0.3. This could not be explained by either a faster proliferation rate or by alterations in cell cycle distribution. Both cell lines were treated with simvastatin, 17-AAG and R115777, agents which inhibit Ras signalling. Cell proliferation was inhibited in both cell lines. The spectrum of NIH 3T3 cells was not affected by treatment. In contrast, in the *ras* transfected cells growth inhibition was associated with an average 35 ± 5% drop in PC levels and a comparable drop in PC/NTP. Thus the MRS visible increase in phosphocholine is associated with Ras activation, and response to treatment is associated with partial reversal of phosphocholine increase in *ras* transfected cells. MRS might therefore be a useful tool in detecting Ras activation and its inhibition following targeted therapies. © 2001 Cancer Research Campaign http://www.bjcancer.com


*British Journal of Cancer* (2001) **84**(5), 691–696
© 2001 Cancer Research Campaign
doi: 10.1054/ bjoc.2000.1663, available online at http://www.idealibrary.com on IDEAL

http://www.bjcancer.com

### Magnetic resonance detects changes in phosphocholine associated with *Ras* activation and inhibition in NIH 3T3 cells

**SM Ronen, LE Jackson, M Beloueche and MO Leach**

Cancer Research Campaign (CRC) Clinical Magnetic Resonance Research Group, Institute of Cancer Research, Royal Marsden Hospital, Downs Road, Sutton, Surrey SM2 5PT, UK

**Summary** *Ras* is frequently mutated in cancer, and novel therapies are being developed to target Ras signalling. To identify non-invasive surrogate markers of Ras activation and inhibition, we used $^{31}P$ magnetic resonance spectroscopy (MRS) and investigated NIH 3T3 cells compared to a mutant *ras* transfected counterpart. The MR spectra indicated that phosphocholine (PC) levels increased significantly from 3 ± 2 fmol cell$^{-1}$ in NIH 3T3 cells to 13 ± 4 fmol cell$^{-1}$ in the transfected cells. The PC/NTP ratio increased significantly from 0.3 ± 0.1 to 0.7 ± 0.3. This could not be explained by either a faster proliferation rate or by alterations in cell cycle distribution. Both cell lines were treated with simvastatin, 17-AAG and R115777, agents which inhibit Ras signalling. Cell proliferation was inhibited in both cell lines. The spectrum of NIH 3T3 cells was not affected by treatment. In contrast, in the *ras* transfected cells growth inhibition was associated with an average 35 ± 5% drop in PC levels and a comparable drop in PC/NTP. Thus the MRS visible increase in phosphocholine is associated with Ras activation, and response to treatment is associated with partial reversal of phosphocholine increase in *ras* transfected cells. MRS might therefore be a useful tool in detecting Ras activation and its inhibition following targeted therapies. © 2001 Cancer Research Campaign http://www.bjcancer.com

**Keywords**: magnetic resonance; $^{31}P$ spectroscopy; phosphocholine; *Ras*; NIH 3T3

Members of the *ras* oncogene family are mutated in a high proportion of human cancers (Kiarkis and Spandidos, 1995), leading to constitutive Ras activation. The overall incidence of *ras* mutations in cancer is of the order of 30% ranging from 80–90% in pancreatic cancers, 30–60% in colorectal cancers and 30–60% in lung cancers. Targeting Ras and the signalling pathways activated by the mutant oncogene is consequently one of the new approaches taken in the development of novel cancer therapies (Boral et al, 1998). Identifying direct or surrogate markers for the efficacy of such treatments could present another challenge. In this context, magnetic resonance spectroscopy (MRS), if able to detect metabolic changes associated with Ras activation or inhibition, could provide a useful non-invasive tool.

$^{1}H$ and $^{31}P$ MRS can be utilized to detect choline, phosphocholine (PC) and glycerophosphocholine (GPC) and to monitor tumour phospholipid metabolism in treated cells and tumours (Podo, 1999 and references therein). At the same time, recent investigations of Ras signalling have demonstrated that the activities of enzymes involved in phosphatidylcholine metabolism, as well as the levels of several choline-containing metabolites are altered following Ras activation. An increase in cellular choline, phosphocholine as well as diacylglycerol (DAG) and GPC have been reported in *ras* transfected NIH 3T3 cells, oocytes, and C3H10T cells (Lacal et al, 1987; Price et al, 1989; Lacal, 1990; Teegarden et al, 1990; Ratnam and Kent, 1995). These variations have been explained by changes in the activities of choline kinase or CTP-PC cytidyltransferase (Teegarden et al, 1990; Ratnam and Kent, 1995; Weiprecht et al, 1996); activation of a phosphatidylcholine (PdylCh) specific phospholipase C (Lopez-Barahona et al, 1990; Carnero et al, 1994; Bjorkoy et al, 1995; Podo et al, 1996); phospholipase D (Carnero et al, 1994) or phospholipase A2 (Lin et al, 1993; Heasley et al, 1997). Using MRS, an increase in PC levels was detected in Schwann cells, following transfection with *H-ras* combined with the SV40 large T antigen (Bhakoo et al, 1996), whereas the comparison of a series of human breast cancer cell lines has shown a correlation between an increasingly transformed phenotype and higher PC/GPC levels (Aboagye and Bhujwalla, 1999).

Using NIH 3T3 cells, a well established model for investigating *ras* transformation, the purpose of this work was to determine whether MRS can be used to detect the alterations in choline metabolism associated with *ras* transformation. Furthermore, we aimed to assess the usefulness of MRS in non-invasively monitoring response to novel therapies targeted at the Ras signalling pathways. By comparing NIH 3T3 cells with their mutant *H-ras* transfected counterparts we determined that MRS can detect a significant increase in PC levels following Ras activation. Subsequent to treatment with 3 different inhibitors of Ras signalling, this increase in PC was partially reversed in the *ras* transfected cell line. We speculate that PC or the PC/NTP ratio could be used as surrogate MRS markers for *ras* transformation and response to Ras targeting therapies.

*Received 11 September 2000*
*Revised 28 November 2000*
*Accepted 7 December 2000*

*Correspondence to:* SM Ronen

### MATERIALS AND METHODS

NIH 3T3 mouse fibroblasts and D12Hras1C (D12H) cells transfected with the *H-ras* gene mutated at codon 12 were kindly provided by C Marshall and L Kelland. Cells were maintained in DMEM (Gibco UK) supplemented with 5% horse serum,

80 U $ml^{-1}$ penicillin, and 80 μg $ml^{-1}$ streptomycin at 37˚C in a 5% $CO_2$ atmosphere.

To characterize cell proliferation, growth curves were determined by counting trypsinized cells every 24 h over 8 days using a Coulter Act 8 counter. Cell cycle analysis was performed on cells fixed in 70% ethanol, treated with 100 μg $ml^{-1}$ RNase A in PBS and stained with 4 μg $ml^{-1}$ propidium iodide, using an Elite ESP Beckman coulter cell sorter at 488 nm. Data were analysed using the WinMdi and Cylchred software (University of Wales College of Medicine).

To determine total protein content, cells were lysed and protein content determined using a DC protein assay kit (Biorad) and measuring absorbance at 750 nm using BSA as standard. Alternatively, the protein precipitate obtained from cells extracted for MRS investigations was dissolved by heating to 60˚C in 1M NaOH and protein content determined as above. To inhibit Ras signalling cells were treated either with 20 μM simvastatin (courtesy of Merck Sharp & Dohme UK; stock solution prepared in DMSO and diluted 1:5000) for 24 h or with 1.3 μM 17-AAG (courtesy of NCI; stock solution in DMSO and diluted 1:5000) for 30 h, or with 1 μM R115777 (courtesy of Janssen; stock solution in water diluted 1:1000) for 72 h. In every case appropriate carrier treated (DMSO or water) controls were used. To assess levels of protein expression ca. $2 \times 10^6$ trypsinized cells were lysed in 50 μl sample buffer (50% buffer II (0.25 M Tris base, 6.9 mM sodium dodecylsulphate (SDS) at pH 6.8), 4% SDS, 2% dithiothreitol, (10% glycerol, 5% 0.1 M phenylmethylsulphonylfluoride and water), and heated to 90˚C for 5 min. Samples were loaded onto a 10% (for ERK, Raf, GAPDH) or 13% (for Ras) polyacrylamide gel, separated by electrophoresis and transferred onto an immobilon-P membrane for 1 h at 80 mA using transfer buffer (15% methanol, 0.3% Tris, 1.44% glycine and water). Immunoblots were blocked overnight in 5% non-fat milk in wash buffer (0.01 M Tris, 0.1 M NaCl at pH 7.5) and probed for 1 h with one of the following: anti-Ras mouse monoclonal antibody (Transduction Laboratories USA); anti-Raf-1 rabbit polyclonal antibody (Santa Cruz Biotechnology USA); anti-ERK rabbit polyclonal antibody (New England Biolabs USA); anti-P-ERK mouse monoclonal antibody (Sigma UK); anti-GAPDH mouse monoclonal antibody (Chemicon International USA). Specific antigen–antibody interactions were detected with horseradish peroxidase-linked secondary antibody using enhanced chemiluminescence Western blotting detection reagents (Amersham Pharmacia Biotech UK).

To obtain an MR spectrum $2–4 \times 10^7$ cells in logarithmic phase were extracted as previously described (Tyagi et al, 1996; Ronen et al, 1999). Briefly, cells were rinsed with ice cold saline, then covered with ice-cold methanol, scraped off the culture flask surface, collected and vortexed. An equal volume of chloroform was then added followed by an equal volume of de-ionized water. Following phase separation and solvent removal samples were stored at –80˚C. Prior to acquisition of the MRS spectra the water soluble metabolites were resuspended in $D_2O$ with 10 mM EDTA at pH 8.2 and the lipids were resuspended in $CDCl_3$ supplemented with methanolic EDTA. MRS spectra were acquired at room temperature on a 400 MHz Bruker spectrometer at 161 MHz using a 90˚ flip angle, a 7 s relaxation delay and broad band proton decoupling during acquisition. Metabolite contents were determined by integration, normalized relative to an internal reference (methylenediphosphonic acid in the case of water soluble metabolites and trimethylphosphate in the case of lipids) and corrected for saturation and the number of cells extracted.

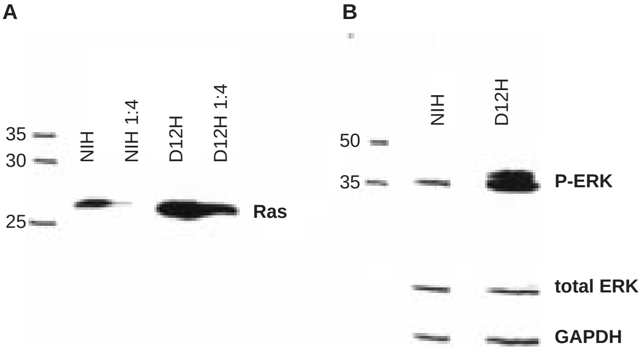

**Figure 1** Western blot analysis determining expression of (**A**) Ras and (**B**) phosphorylated ERK and total ERK proteins in NIH 3T3 (NIH) cells and D12H *ras*-transfected cells. GAPDH was used to confirm equal sample loading. Lanes marked 1:4 were loaded with sample diluted 1:4 relative to the neighbouring lane

Results represent an average of at least 3 repetitions and are expressed as mean ± SD. Statistical significance was determined using one way ANOVA (Arcus Quickstat – Longman Software Publishing) and differences were assumed to be significant when $P < 0.05$.

### RESULTS

Western blots confirmed that Ras levels were higher in the *ras* transfected D12H cell line (Figure 1A). To determine the level of signalling through the Ras-Raf-MEK-ERK pathway the levels of both total ERK, and the phosphorylated form of ERK were determined by Western blotting. As illustrated in Figure 1B the levels of phosphorylated ERK were higher in the *ras* transfected D12H cells whereas total ERK levels indicate that the overall protein levels were not substantially altered following transfection. Western blotting for GAPDH was used to confirm equal loading of samples.

Cell growth was characterized by determining the proliferation rate of the cells as well as the cell cycle distribution. We determined that the doubling time was 28 ± 4 hours for NIH 3T3 cells and 32 ± 4 hours for D12H cells ($n = 8$, $P = 0.07$). The cell cycle distribution of exponentially growing NIH 3T3 cells was 64 ± 8% in G1, 7 ± 3% in G2 and 29 ± 6% in S phase. In the D12H cells the cell cycle distribution was altered: 40 ± 4% of cells were in $G_1$, 20 ± 5% of cells were in $G_2$ and 40 ± 5% of cells were in S phase during the mid-logarithmic phase ($P < 0.03$ for $G_1$).

Protein content was determined from cells extracted for this purpose and confirmed from protein precipitates obtained following cell extractions for MRS. The protein content of NIH 3T3 cells was 249 ± 28 pg $cell^{-1}$ and increased significantly to 391 ± 61 pg $cell^{-1}$ in D12H cells ($P < 0.001$).

Figure 2 illustrates the $^{31}P$ MRS spectra obtained from extracts of NIH 3T3 and D12H cells. To rule out the effects of cell cycle arrest due to confluence, care was taken to perform all investigations when cells were in mid-logarithmic phase. The average PC content of NIH 3T3 cells was 3 ± 2 fmol $cell^{-1}$ ($n = 5$) significantly increasing to 13 ± 4 fmol $cell^{-1}$ ($n = 5$) in the D12H cells ($P < 0.001$). Cellular NTP content also increased upon *ras* transfection from 13 ± 3 fmol $cell^{-1}$ to 20 ± 7 fmol $cell^{-1}$ ($P < 0.04$). The PC/NTP ratio was 0.3 ± 0.1 in NIH 3T3 cells and increased significantly to 0.7 ± 0.3 in the D12H cells ($P < 0.001$). The GPC signal

© 2001 Cancer Research Campaign

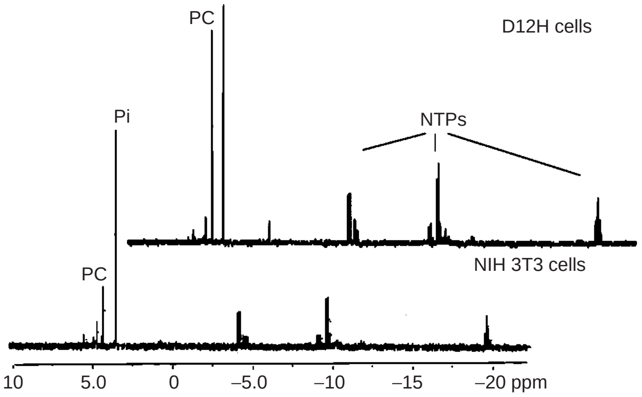

**Figure 2** $^{31}$P MRS spectra of the water soluble metabolites obtained from NIH 3T3 and D12H cell extracts. The spectrum was plotted with a line broadening of 0.2Hz. PC: phosphocholine: Pi: inorganic phosphate; NTP: nucleoside triphosphates

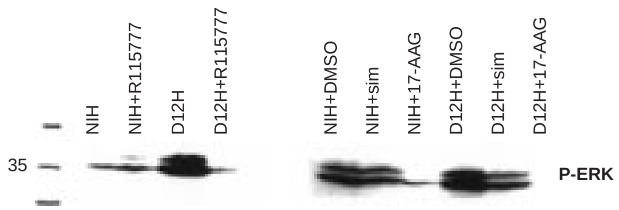

**Figure 3** Western blot analysis determining expression of phosphorylated ERK in NIH 3T3 (NIH) and D12H cells treated with R115777, simvastatin (sim), and 17-AAG compared to matched controls was not always detectable by MRS and no statistically significant differences between the two cell lines could be observed.

To determine whether the levels of phosphatidylcholine were also altered following *ras* transformation, spectra were recorded from the chloroform phase of the cell extracts. No statistically significant difference between NIH 3T3 and D12H cells was observed. Phosphatidylcholine represented 48 ± 13% of total phospholipid content in NIH 3T3 cells and 49 ± 3% in D12H cells. This indicated that any change in the phospholipid content is below detection level by MRS, and cellular phosphatidylcholine level was not substantially affected by the increase in its precursor phosphocholine.

To further ascertain that our observations were correlated with the activation of Ras, and to determine whether MRS could be used to assess response to Ras targeting therapies, we treated both cell lines with 3 different inhibitors of the Ras pathway – R115777, simvastatin and 17-AAG. R115777 is an inhibitor of the farnesyl-transferase enzyme which catalyses binding of a farnesyl moiety to the Ras protein (the farnesyl is required for localization of Ras to the cell membrane) (Zujewski et al, 2000). Simvastatin is an HMG-CoA reductase inhibitor which inhibits synthesis of the farnesyl moiety (Leonard et al, 1990). 17-AAG is an HSP90-binding protein and causes depletion of several proteins including Raf, downstream of Ras in signalling to ERK (Schulte and Necker, 1998). Figure 3 illustrates the results of Western blotting for phosphorylated ERK. As indicated by the drop in P-ERK levels, all 3 inhibitors caused an inhibition of signalling through the Ras-Raf-MEK-ERK pathway. This inhibition was more marked in the case of D12H cells. Following treatment with 17-AAG a substantial drop in total Raf levels was also observed in both cell lines but total ERK was not altered following any of the treatments (data not shown).

Treatment with the Ras inhibitors also inhibited cell proliferation as illustrated in Figures 4A and 4E (NIH 3T3 and D12H cells respectively). In the case of NIH 3T3 cells, the number of cells per flask dropped to 72 ± 15% following R115777 treatment ($P < 0.03$), 61 ± 15% following simvastatin treatment ($P < 0.03$), and 24 ± 16% following 17-AAG treatment ($P < 0.01$) relative to control. In the case of D12H cells the number of cells per flask dropped to 62 ± 15% following simvastatin treatment ($P < 0.05$), and to 32 ± 4% following 17-AAG treatment ($P < 0.005$) relative to control. Following treatment with R115777, a drop in cell number per flask was observed but was not statistically significant (down to 88 ± 12%, $P = 0.2$).

Treatment affected the cell cycle distribution in the same manner in both cell lines. R115777 and 17-AAG did not significantly alter the distribution in either cell line. In contrast simvastatin caused an arrest in the $G_1$ phase of the cycle in both lines. In NIH 3T3 cells, the cell cycle distribution following treatment was 83 ± 4% in $G_1$, 4 ± 1% in $G_2$ and 13 ± 4% in S, and in the D12H cells the distribution was 77 ± 4% in $G_1$, 7 ± 2% in $G_2$ and 16 ± 2% in S phase. Total protein content was not significantly affected by treatment in either cell line.

The MRS spectra of treated and control cell extracts indicated that for NIH 3T3 cells, the inhibition of Ras signalling and cell growth was not accompanied by any significant changes in the metabolites present in the $^{31}$P MRS spectrum (Figure 4B–D). Within experimental error PC and NTP levels remained constant following all 3 treatments. In contrast, in the case of D12H *ras*-transfected cells, the inhibition in cell growth and Ras signalling were accompanied by a significant drop in the levels of PC (Figure 4F). PC content dropped from 17 ± 5 in DMSO-treated controls to 11 ± 1 fmol cell$^{-1}$ following treatment with simvastatin ($P < 0.02$) and to 11 ± 1 fmol cell$^{-1}$ following treatment with 17-AAG ($P < 0.01$). The increase observed in PC levels in the DMSO treated controls versus untreated control (13 ± 3 versus 17 ± 5) was not statistically significant ($P = 0.3$) The levels of NTP in the treated D12H cells remained unchanged within experimental error (Figure 4G) thus PC/NTP (Figure 4H) dropped significantly from 0.8 ± 0.1 in DMSO treated controls to 0.4 ± 0.1 following treatment with 17-AAG ($P < 0.04$) and to 0.6 ± 0.1 following simvastatin treatment ($P < 0.03$). Treatment with R115777 was also accompanied by a drop in PC from 13 ± 4 to 8 ± 6 fmol cell$^{-1}$, as well as a drop in PC/NTP from 0.7 ± 0.3 to 0.4 ± 0.1 but these did not reach statistical significance ($P = 0.06$).

### DISCUSSION

To assess the effect of Ras activation and its inhibition using MRS we chose to concentrate on the extensively studied NIH 3T3 mouse fibroblast line and a mutant *ras*-transfected counterpart. By Western blotting for Ras and phosphorylated ERK, we confirmed that Ras signalling was increased in the transfected D12H cells relative to the parent NIH 3T3 line. The MRS investigations of cell extracts showed a significant difference in metabolite content following *ras* transfection. NTP levels were higher in the *ras* transfected cells, but the most significant difference was in phosphocholine content which was more than 4 fold higher per cell in D12H. If metabolite content was normalized to mg protein, NTP content was unaltered by Ras (both cell lines contain

© 2001 Cancer Research Campaign

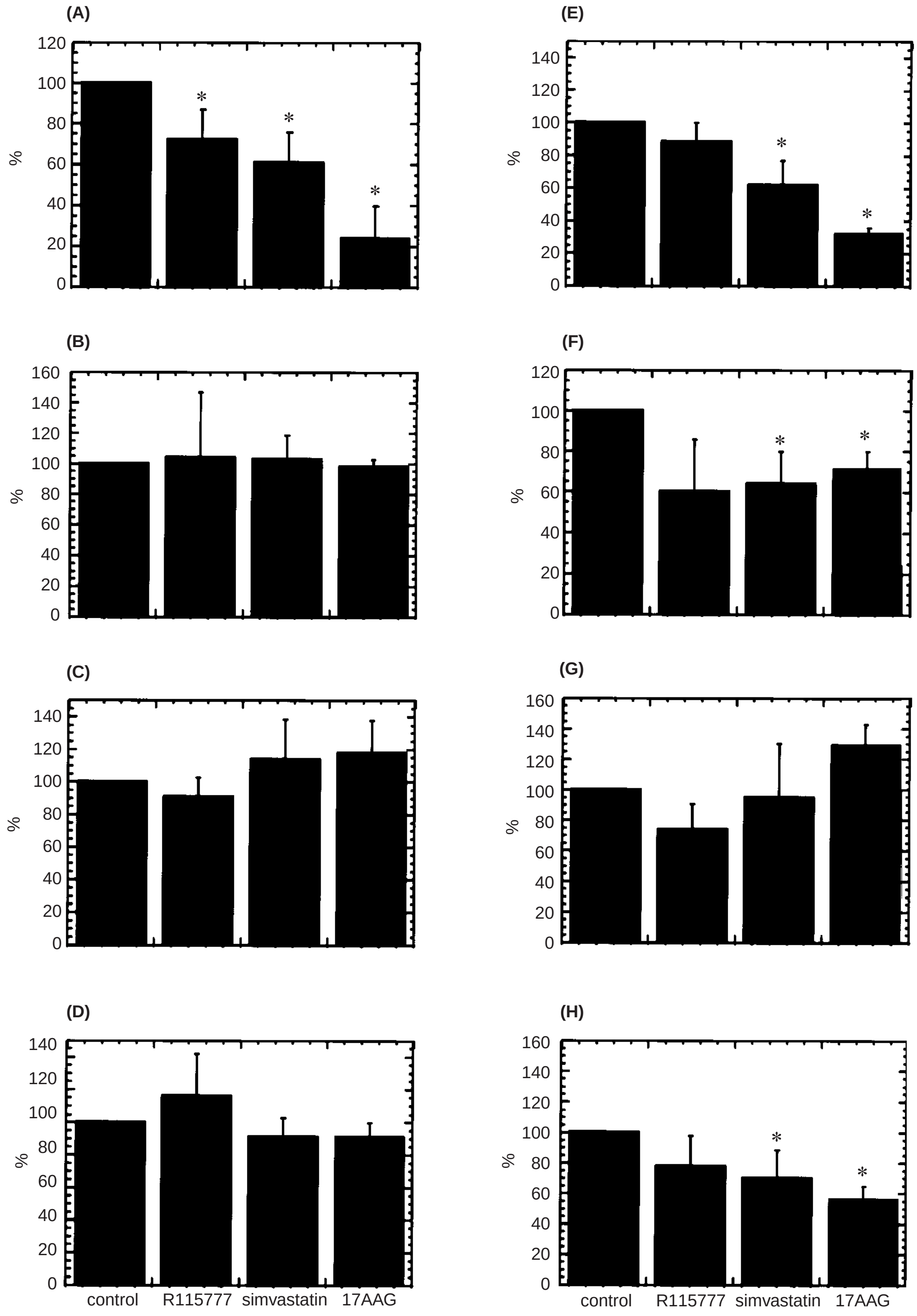

**Figure 4** NIH 3T3 cells **(A–D)** and D12H cells (E–H) following treatment with R115777, simvastatin and 17-AAG. **A** and **E** represent % cell number per flask following treatment relative to control. **B** and **F** represent % PC content per cell relative to control. **C** and **G** represent % NTP content per cell relative to control. **D** and **H** represent % PC/NTP content per cell relative to control. Results represent the average change of 3 experiments relative to their matched controls (not the relative change in average values). * Signifies statistical significance ($P < 0.05$)

5 nmoles $mg^{-1}$ protein). However, PC levels showed an almost 3-fold increase from 12 nmol $mg^{-1}$ protein in the NIH 3T3 cell to 33 nmol $mg^{-1}$ protein in the D12H line. The average PC/NTP ratio, a parameter independent of cell number or protein content, also increased significantly by more than 2-fold indicating that this ratio could serve as an MRS marker of Ras activation.

Previous MRS work has shown that PC levels can be affected by the proliferation state of the cells, with a drop in PC content observed in quiescent cells (Ronen et al, 1992; Podo, 1999). To avoid this problem care was taken to compare cells which were in the logarithmic phase of their growth, excluding cell populations which could be arrested due to confluence or nutrient deprivation.

 *© 2001 Cancer Research Campaign*

The increase in PC levels in transformed cells and tumours has been postulated as resulting from an increase in phospholipid synthesis to produce membranes required during cell division (Negendank, 1992; Podo, 1999). Our results show that the -*ras*-transfected cell line which has a higher PC content, has a doubling time comparable within experimental error to the doubling time of the control cells. Thus, in this model, PC levels were not associated with altered doubling time. This observation is in agreement with some previously published data (Ting et al, 1996; Aboagye and Bhujwalla, 1999).

Previous MRS work has also shown a correlation between S phase fraction and PC levels (Smith et al, 1991). We observed a shortening of the $G_1$ phase of the cell cycle in the *ras*-transfected D12H cells. This probably results from the control by Ras of cyclin D (Liu et al, 1995; Gille and Downward, 1999). We cannot rule out that in our cells this drop in $G_1$ contributes to the observed increase in PC. However this is probably not the only explanation for our results: even if PC levels were undetectable during the $G_1$ phase with PC originating exclusively from cells in $G_2$ + S, the observed increase in the $G_2$ + S fraction from 36% to 60% following *ras* transfection would not be sufficient to explain the 4-fold increase in PC content per cell observed in the D12H cells.

To further confirm that the increase in PC levels was due to Ras activation and to assess whether MRS could be used to detect the effects of Ras targeting therapies we treated both cell lines with 3 inhibitors of the Ras signalling pathway. We chose 2 inhibitors which would affect all pathways downstream of Ras (simvastatin and R115777) and a third inhibitor which should primarily affect the Ras-Raf-MEK-ERK pathway by depleting Raf (17-AAG). Whereas the spectrum of NIH 3T3 cells was not affected by treatment, D12H cells showed a drop in PC levels after treatment. The 3 inhibitors affected cell viability and cell cycle distribution of the control and transfected cell lines in the same way, ruling out differential response to treatment as an explanation of our results. Our findings lead to the conclusion that the MR detectable drop in PC is associated with inhibition in Ras signalling and cell proliferation only in the cells which over-expressed *ras*. This indicates that the initial increase in PC and PC/NTP and its subsequent partial drop following treatment probably reflect the presence of the mutant activated Ras protein.

Our results require further confirmation in other models, including human cell lines expressing mutant Ras. Nonetheless, this study indicates that MRS could be useful in non-invasively monitoring Ras activation as well as response to novel, Ras targeting therapies, with phosphocholine and PC/NTP levels serving as surrogate markers for mutant Ras activity.

### ACKNOWLEDGEMENTS

CRC funding is gratefully acknowledged by SMR and MOL (grant number SP SP1780/0103).

### REFERENCES

Aboagye EO and Bhujwalla ZM (1999) Malignant transformation alters membrane choline phospholipid metabolism of human mammary epithelial cells. *Cancer Res* **59**: 80–84

Bhakoo KK, Williams SR, Florian CL, Land H and Noble MD (1996) Immortalization and transformation are associated with specific alternations in choline metabolism. *Cancer Res* **56**: 4630–4634

Bjorkoy G, Overvatn A, Diaz-Meco MT, Moscat J and Johansen T (1995) Evidence for a bifurcation of the mitogenic signaling pathway activated by ras and phosphatidylcholine-hydrolyzing phsopholipase C. *J Biol Chem* **270**: 21299–21306

Boral AL, Dessain S and Chabner BA (1998) Clinical evaluation of biologically targeted drugs: obstacles and opportunities. *Cancer Chemother Pharmacol* **42**: s3–s21

Carnero A, Cuadrado A, Del Peso L and Lacal JC (1994) Activation of D phospholipase by serum stimulation and ras-induced transformation in NIH3T3 cells. *Oncogene* **9**: 1387–1395

Gille H and Downward J (1999) Multiple ras effector pathways contribute to G1 cell cycle progression. *J Biol Chem* **274**: 22033–22040

Heasley LE, Thaler S, Nicks M, Price B, Skorecki K and Nemenoff RA (1997) Induction of cytosolic phospholipase A2 by oncogenic Ras in human non-small cell lung cancer. *J Biol Chem* **272**: 14501–14504

Kiarkis H and Spandidos DA (1995) Mutations of ras genes in human tumours (review). *Int J Oncol* **7**: 413–421

Lacal JC (1990) Diacylglycerol production in *Xenopus laevis* oocytes after microinjection of p21ras proteins is a consequence of activation of phosphatidylcholine metabolism. *Mol Cell Biol* **10**: 333–340

Lacal JC, Moscat J and Aaronson SA (1987) Novel source of 1,2-diacylglycerol elevated in cells transformed by Ha-ras oncogene. *Nature* **330**: 269–272

Leonard S, Beck L and Sinensky M (1990) Inhibition of isoprenoid biosynthesis and the post-translational modification of pro-p21. *J Biol Chem* **265**: 5157–5160

Lin LL, Wartmann M, Lin AY, Knopf JL, Seth A and Davis RJ (1993) cPlA2 is phosphorylated and activated by MAP kinase. *Cell* **72**: 269–278

Liu JJ, Chao JR, Ming-Chung J, Sun-Yu N, Yen JJY and Yang-Yen HF (1995) Ras transformation results in an elevated level of cyclin D1 and accelerationof G1 progression in NIH 3T3 cells. *Mol Cell Biol* **15**: 3654–3663

Lopez-Barahona M, Kaplan PL, Cornet ME, Diaz Meco MT, Larrodera P, Diaz-Laviada I, Municiao AM and Moscat J (1990) Kinetic evidence of a rapid activation of phosphatidylcholine hydrolysis by Ki-ras oncogene. Possible involvement in late steps of mitogenic cascade. *J Biol Chem* **265**: 9022–9026

Negendank W (1992) Studies of human tumours by MRS: a review. *NMR Biomed* **5**: 303–324

Podo F (1999) Tumour phospholipid metabolism. *NMR in Biomed* **12**: 413–439

Podo F, Ferretti A, Knijn A, Zhang P, Ramoni C, Barletta B, Pini C, Baccarini S and Pulciani S (1996) Detection of phosphatidylcholine-specific phospholipase C in NIH-3T3 fibroblasts and their H-ras transformants: NMR and immunochemical studies. *Anticancer Res* **16**: 1399–1412

Price BD, Morris JDH, Marshall CJ and Hall A (1989) Stimulation of phosphatidylcholine hydrolysis, diacylglycerol release and arachidonic acid production by oncogenic ras is a consequence of protein kinase C activation. *J Biol Chem* **264**: 16638–16643

Ratnam S and Kent C (1995) Early increase in choline kinase activity upon induction of the H-ras oncogene in mourse fibroblast cell lines. *Arch Biochem Biophys* **323**: 313–322

Ronen SM, Rushkin E and Degani H (1992) Lipid metabolism in large T47D human breast cancer spheroids: 31P- and 13C-NMR studies of choline and ethanolamine uptake. *Biochim Biophys Acta* **1138**: 203–212

Ronen SM, DiStefano F, McCoy CL, Robertson D, Smith TAD, Al-Saffar NM, Titley J, Cunningham DC, Griffiths JR, Leach MO and Clarke PA (1999) Magnetic resonance detects metabolic changes associated with chemotherapy-induced apoptosis. *Br J Cancer* **80**: 1035–1041

Schulte TW and Necker LM (1998) The benzoquinone ansamycin 17-allylamino-17-demethoxygeldanamycin binds to HSP90 and shares important biologic activities with geldanamycin. *Cancer Chemother Pharmacol* **42**: 273–279

Smith TA, Eccles S, Ormerod MG, Tombs AJ, Titley JC and Leach MO (1991) The phosphocholine and glycerophosphocholine content of an oestrogen-sensitive rat mammary tumour correlates strongly with growth rate. *Br J Cancer* **64**: 821–826

Teegarden D, Taparowsky EJ and Kent C (1990) Altered phosphatidylcholine metabolism in C3H10T cells transfected with the Harvey-ras oncogene. *J Biol Chem* **265**: 6042–6047

Ting Y-LT, Sherr D and Degani H (1996) Variations in energy and phosphlipid metabolism in normal and cancer human mammary epithelial cells. *Anticancer Res* **16**: 1381–1388

Tyagi RK, Azrad A, Degani H and Salomon Y (1996) Simultaneous extraction of cellular lipids and water-soluble metabolities: evaluation by NMR spectroscopy. *Magn Reson Med* **35**: 194–200

© 2001 Cancer Research Campaign 

Weiprecht M, Weider T, Paul C, Geilen CC and Orfanos CE (1996) Evidence for phosphorylation of CTP:phosphocholine cytidyltrasferase by multiple proline directed protein kinases. *J Biol Chem* **271**: 9955–9961
Zujewski J, Horak ID, Bol CJ, Woestenborghs R, Bowden C, End DW, Piotrovsky VK, Chiao J, Belly RT, Todd A, Kopp WC, Kohler DR, Chow C, Noone M, Hakim FT, Larkin G, Gress RE, Nussenblatt RB, Kremer AB and Cowan KH (2000) Phase I and Pharmacokinetic Study of Farnesyl Protein Transferase Inhibitor R115777 in Advanced Cancer. *J Clin Oncol* **18**: 927–928

*© 2001 Cancer Research Campaign*
